# Defect Chemistry and Na-Ion Diffusion in Na_3_Fe_2_(PO_4_)_3_ Cathode Material

**DOI:** 10.3390/ma12081348

**Published:** 2019-04-25

**Authors:** Navaratnarajah Kuganathan, Alexander Chroneos

**Affiliations:** 1 Department of Materials, Imperial College London, London SW7 2AZ, UK; alexander.chroneos@imperial.ac.uk; 2 Faculty of Engineering, Environment and Computing, Coventry University, Priory Street, Coventry CV1 5FB, UK

**Keywords:** Na_3_Fe_2_(PO_4_)_3_, defects, Na-ion diffusion, dopant, atomistic simulation

## Abstract

In this work, we employ computational modeling techniques to study the defect chemistry, Na ion diffusion paths, and dopant properties in sodium iron phosphate [Na_3_Fe_2_(PO_4_)_3_] cathode material. The lowest intrinsic defect energy process (0.45 eV/defect) is calculated to be the Na Frenkel, which ensures the formation of Na vacancies required for the vacancy-assisted Na ion diffusion. A small percentage of Na-Fe anti-site defects would be expected in Na_3_Fe_2_(PO_4_)_3_ at high temperatures. Long-range diffusion of Na is found to be low and its activation energy is calculated to be 0.45 eV. Isovalent dopants Sc, La, Gd, and Y on the Fe site are exoergic, meaning that they can be substituted experimentally and should be examined further. The formation of Na vacancies and Na interstitials in this material can be facilitated by doping with Zr on the Fe site and Si on the P site, respectively.

## 1. Introduction

Rechargeable sodium ion batteries (SIBs) have gained considerable attention for the development of large-scale energy storage applications due to the high abundance, low cost, and non-toxicity of sodium [1,2,3,4,5]. In practice, there are only a few electrode materials that have been reported. This is due to the larger ionic radius of Na compared to that of Li. Designing a new Na-based electrode material consisting of an appropriate transition metal with a high electrochemical performance could make this material promising.

A number of iron-based phosphate cathode materials [6,7,8,9,10] were proposed for Li-ion batteries due to the structural stability and high redox potential provided by the PO_4_^3^^−^ matrix. Although similar iron-based cathode materials can be prepared for NIBs in theory, only a few of them, including NaFePO_4_ [11], Na_2_FeP_2_O_7_ [12], Na_3_V_2_(PO_4_)_3_ [13] and Na_4_Fe_3_(PO_4_)_2_P_2_O_7_ [14], have been reported in the literature.

Recently, monoclinic phase Na_3_Fe_2_(PO_4_)_3_ was synthesized using the solid state method and examined as a cathode material for SIBs [15]. This material showed a very high cyclic stability and a reversible discharge capacity of 40 mA·h·g^−1^ with a flat plateau at about 2.5 V. Rajagopalan et al. [16] studied the electrochemical performance and reversible capacity of Na_3_Fe_2_(PO_4_)_3_. Their study shows that the discharge specific capacity and cycling stability can be improved by wrapping Na_3_Fe_2_(PO_4_)_3_ with conducting carbon. There are no further experimental or theoretical studies available on Na_3_Fe_2_(PO_4_)_3_ for the use of this material in rechargeable SIBs.

A fundamental understanding of Na_3_Fe_2_(PO_4_)_3_ gained through computational simulation techniques based on the classical pair-potentials can be used to optimize its performance as these techniques have been useful in experimental characterization, the prediction of ion pathways, and the determination of promising dopants. This technique has been used in a variety of oxide materials, including electrode materials for lithium and sodium ion batteries [17,18,19,20,21,22,23,24,25,26,27,28,29,30,31,32,33,34,35,36,37,38]. In this work, we have used classical pair-potential simulation to examine possible defects that can be observed in Na_3_Fe_2_(PO_4_)_3_, Na ion migration pathways and the solution of trivalent dopants (Al^3+^, Ga^3+^, Sc^3+^, Y^3+^, Gd^3+^ and La^3+^) on the Fe site and tetravalent dopants (Si^4+^, Ge^4+^, Ti^4+^, Sn^4+^, Zr^4+^ and Ce^4+^) on the Fe and P sites.

## 2. Computational Methods

The calculations employed in this study are based on the classical pair wise potentials. The General Utility Lattice Program (GULP) [39] was used. This code uses ion-ion interactions in the form of long-range (i.e., Coulombic) attraction and short-range repulsion (i.e., Pauli electron-electron) and attraction (dispersion). We used the Buckingham potentials (refer to Table S1 in the Supplementary Information) to model short-range interaction. 

Bulk Na_3_Fe_2_(PO_4_)_3_ and defect configurations were optimized using the Broyden-Fletcher-Goldfarb-Shanno (BFGS) algorithm [40], as implemented in the GULP code. The forces on the atoms were below 0.001 eV/Å in all cases. The Mott-Littleton method [41] was employed to model point defects. This methodology has been well-explained in previous studies. The current simulation is expected to overestimate the defect enthalpies. This is due to the spherical shape of the ions with a low concentration. Nevertheless, trends in relative energies are expected to be consistent.

The present atomistic simulations use isobaric parameters in the calculations of formation and migration energies. The detailed thermodynamic relations associated with isobaric parameters are discussed in previous theoretical work [42,43,44,45,46,47].

## 3. Results and Discussion

### 3.1. Bulk Na_3_Fe_2_(PO_4_)_3_ Structure

**Figure 1:**
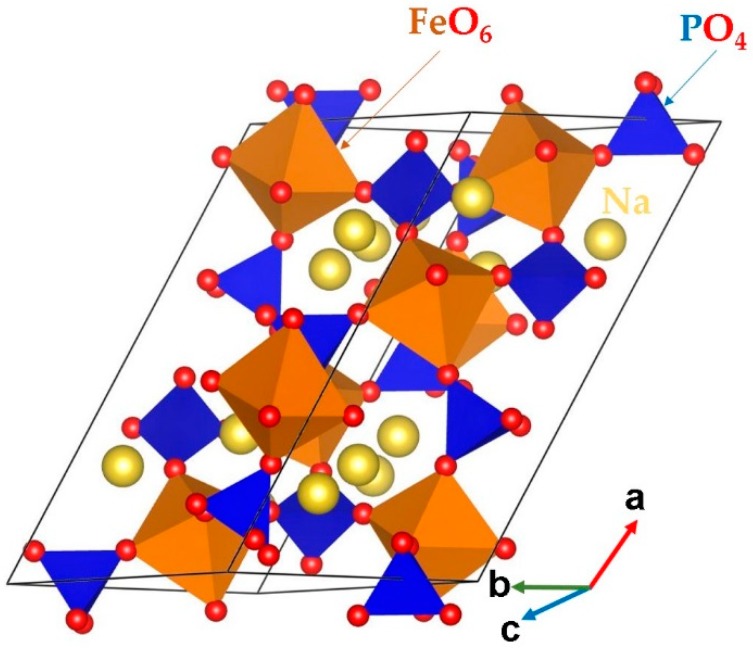
Crystal structure of Na_3_Fe_2_(PO_4_)_3_ (space group *C*2/*c*).

Bulk Na_3_Fe_2_ (PO_4_)_3_ belongs to the monoclinic crystal system (space group *C*2/*c*). The crystal structure of Na_3_Fe_2_(PO_4_)_3_ is shown in Figure 1. Its lattice parameters (*a* = 15.070 Å, *b* = 8.740 Å, *c* = 8.724 Å, *α* = *γ* = 90.0° and *β* = 125.1°) were determined by Fanjet et al. [48] in their powder neutron diffraction study. The crystal structure consists of FeO_6_ octahedra and PO_4_ tetrahedra units sharing their corners.

In order to validate the interatomic potentials used in this study, a bulk Na_3_Fe_2_(PO_4_)_3_ structure was optimized under constant pressure. The difference between the calculated equilibrium lattice constants and its corresponding experimental values is less than 1.5%, showing the good reproduction and the suitability of these potential parameters for defect modeling. Table 1 lists the calculated and experimental values together with the error percentages.

### 3.2. Intrinsic Defect Process

Here, we consider the formation of point defects (vacancies and interstitials) to calculate the Schottky and Frenkel defect processes. The Na-Fe anti-site defect process is also considered. The intrinsic point defects are important as they stimulate the ions to diffuse in the lattice. The point defects were combined to construct reaction energy processes (Schottky, Frenkel and anti-site) using Kröger-Vink notation [49]. The reaction equations are as follows:

(1) $$\mathrm{Na}\;\mathrm{Frenkel}:{\;\mathrm{Na}}_{\mathrm{Na}}^{X}\;\to \;V_{\mathrm{Na}}^{\prime }\;+{\;\mathrm{Na}}_{i}^{•}$$

(2) $$\mathrm{Fe}\;\mathrm{Frenkel}:{\;\mathrm{Fe}}_{\mathrm{Fe}}^{X}\;\to \;V_{\mathrm{Fe}}^{\prime \prime \prime }\;+{\;\mathrm{Fe}}_{i}^{•••}$$

(3) $$P\;\mathrm{Frenkel}:{\;P}_{P}^{X}\;\to \;V_{P}^{\prime \prime \prime \prime \prime }\;+{\;P}_{i}^{•••••}$$

(4) $$O\;\mathrm{Frenkel}:{\;O}_{O}^{X}\;\to \;V_{O}^{••}\;+{\;O}_{i}^{\prime \prime }$$

(5) $$\mathrm{Schottky}:3{\mathrm{Na}}_{\mathrm{Na}}^{X}\;+\;2{\mathrm{Fe}}_{\mathrm{Fe}}^{X}\;+\;3P_{P}^{X}\;+\;12O_{O}^{X}\;\to \;3V_{\mathrm{Na}}^{\prime }\;+\;2V_{\mathrm{Fe}}^{\prime \prime \prime }\;+\;3V_{P}^{\prime \prime \prime \prime \prime }\;+\;12V_{O}^{••}\;+{\;\mathrm{Na}}_{3}{\mathrm{Fe}}_{2}{\left({\mathrm{PO}}_{4}\right)}_{3}$$

(6) $${\mathrm{Na}}_{2}O\;\mathrm{Schottky}:\;2{\mathrm{Na}}_{\mathrm{Na}}^{X}\;+{\;O}_{O}^{X}\;\to \;2V_{\mathrm{Na}}^{\prime }\;+\;V_{O}^{••}\;+{\;\mathrm{Na}}_{2}O$$

(7) $$\mathrm{Na}/\mathrm{Fe}\;\mathrm{antisite}\;\left(\mathrm{isolated}\right):{\;\mathrm{Na}}_{\mathrm{Na}}^{X}\;+{\;\mathrm{Fe}}_{\mathrm{Fe}}^{X}\;\to {\;\mathrm{Na}}_{\mathrm{Fe}}^{\prime \prime }\;+{\;\mathrm{Fe}}_{\mathrm{Na}}^{••}$$

(8) $$\mathrm{Na}/\mathrm{Fe}\;\mathrm{antisite}\;\left(\mathrm{cluster}\right):{\;\mathrm{Na}}_{\mathrm{Na}}^{X}\;+{\;\mathrm{Fe}}_{\mathrm{Fe}}^{X}\;\to \;\{{\mathrm{Na}}_{\mathrm{Fe}}^{\prime \prime }:{\mathrm{Fe}}_{\mathrm{Na}}^{••}{\text{\}}}^{X}$$

**Figure 2:**
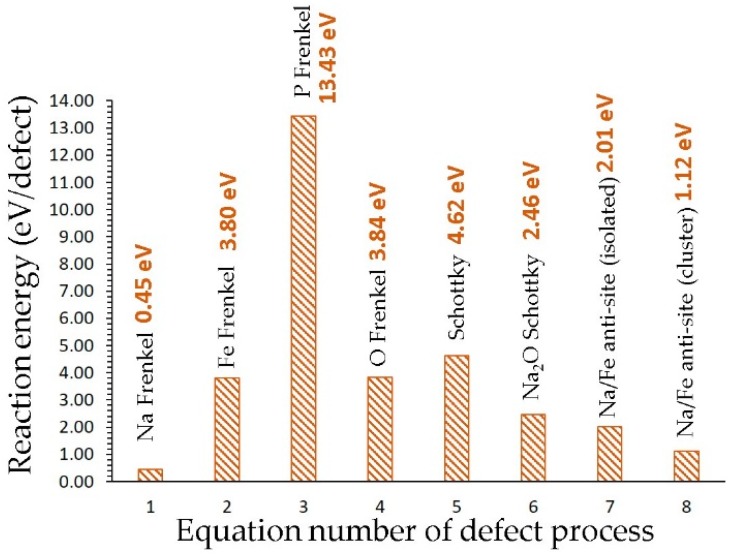
Energetics of intrinsic defect processes.

The energetics for the defect processes are shown in Figure 2. Our calculations show that the Na Frenkel is the lowest defect energy process (0.45 eV/defect), indicating that the formation of Na vacancies is facilitated by this process. Thus, this process would accelerate the vacancy-aided Na diffusion. The Na-Fe anti-site defect is found to be the second most favorable defect energy process (1.12 eV/defect). In this defect, a small population of Na on the Fe site and Fe on the Na site would be observed. This defect has been found in a variety of battery materials both experimentally and theoretically [17,18,19,50,51,52,53]. The formation of Na_2_O is calculated to be a 2.46 eV/defect, suggesting that there is a possibility of Na_2_O loss in this material at high temperatures. Other defect processes are relatively high energy, meaning that they cannot be observed under standard battery operating conditions.

### 3.3. Sodium Ion Diffusion

**Figure 3:**
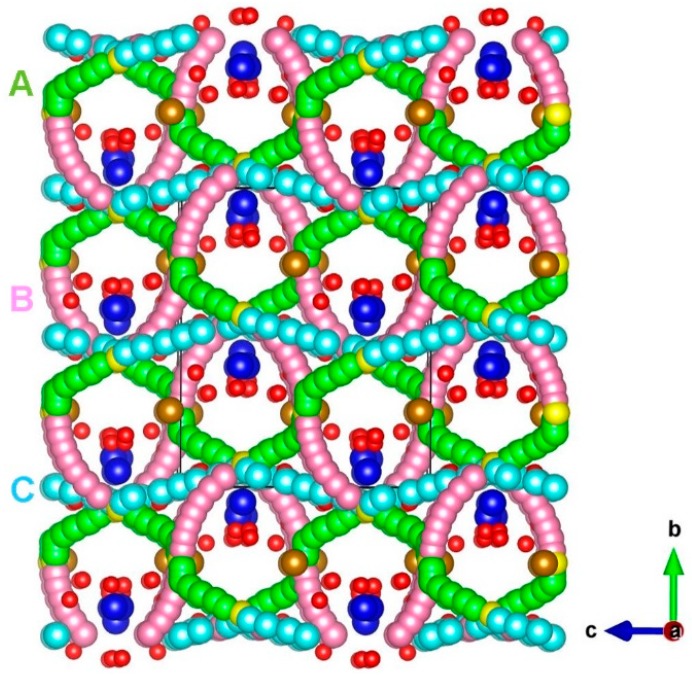
Na ion diffusion paths calculated in Na_3_Fe_2_(PO_4_)_3_. Green, purple, and light blue atoms correspond to different local Na ion hopping trajectories.

**Figure 4:**
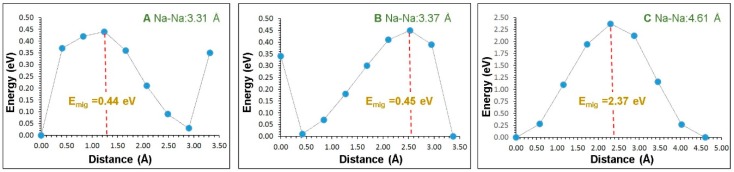
Three different energy profile diagrams for Na local hops, as shown in Figure 3. (**A**–**C**) correspond to the local Na–Na hops.

The diffusion of Na ions with a low migration barrier is a key requirement for a promising cathode material. There is no experimental report on the Na ion migration pathways in Na_3_Fe_2_(PO_4_)_3_. Using the current methodology, it is possible to calculate the Na ion diffusion pathways and activation energies. Three different local Na vacancy migration hops (refer to Figure 3) were calculated. Table 2 lists the Na-Na separations together with the activation energies. Energy profile diagrams together with activation energies for local Na hops are shown in Figure 4. The lowest activation energy (0.44 eV) is calculated for hop A in which Na migrates along the *bc* plane. Hop B exhibits an activation energy of 0.45 eV, which is very close to the value calculated for hop B. The highest activation energy (2.37 eV) is calculated for hop C. Both hops B and C are also along the *bc* plane.

We examined possible long-range Na ion pathways linked by local Na hops. Three possible long-range Na ion paths and their corresponding overall activation energies are listed in Table 3. The lowest activation energy of the migration (0.45) pathway consists of local hops A and B (A–A–B–B) along the *bc* plane. The other two pathways have higher activation energies of 2.37 eV due to the presence of local hop C.

Clark et al. [54] used classical atomistic simulation to calculate the activation energy of Na ions in Na_2_FeP_2_O_7_. In their study, three-dimensional long-range Na ion migration paths were observed in different directions with activation energies in the range of 0.33–0.49 eV. Sodium ion migration paths together with activation energies were calculated in “olivine” NaFePO_4_ material [55]. The lowest energy path was observed along the [010] direction with the activation energy of 0.30 eV. The current activation energy value of 0.45 eV calculated for long-range Na ion migration in Na_3_Fe_2_(PO_4_)_3_ suggests that this material is also a promising cathode material competitive with the other Fe-based polyanions materials for Na-ion batteries.

### 3.4. Isovalent Doping

Solutions of isovalent dopants (Al, Ga, Sc, Y, Gd, and La) were considered on the Fe site. The selection of trivalent dopants with a wide range of ionic radii is based on previous studies that considered these dopants in different materials, including phosphate-based battery materials [23,56,57,58]. Though they exhibit a high atomic weight, low abundance, or high cost, low-level doping of Na_3_Fe_2_(PO_4_)_3_ can exhibit improvement in electronic conductivity. The solution enthalpy was calculated using the following reaction:

(9) $$R_{2}O_{3}\;+\;2{\mathrm{Fe}}_{\mathrm{Fe}}^{X}\;\to \;2R_{\mathrm{Fe}}^{X}\;+\;2{\mathrm{Fe}}_{2}O_{3}$$

**Figure 5:**
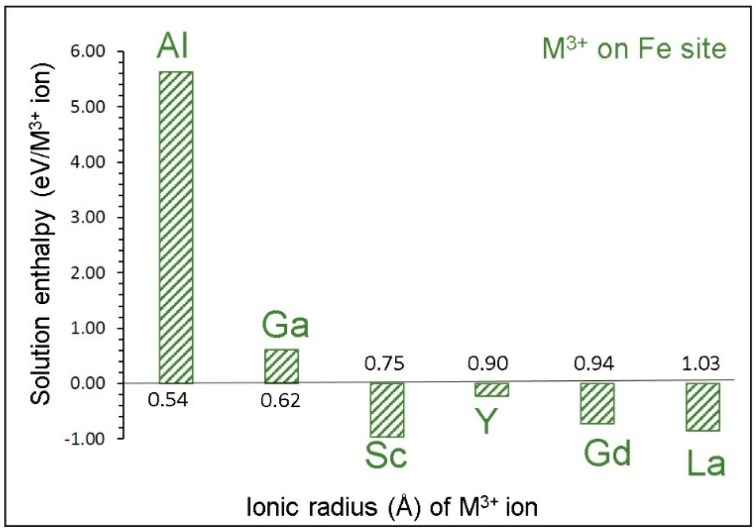
Solution enthalpies calculated for R_2_O_3_ (R= Al, Ga, Sc, Y, Gd and La) with respect to the M^3+^ radius in an octahedral coordination.

Figure 5 reports the solution enthalpies of M^3+^ ions on the Fe site. The lowest solution energy is calculated for Sc. Solutions of La and Gd are also promising as they exhibit negative values. Yttrium shows a small but negative solution enthalpy. The solution enthalpy of Al is highly endoergic, suggesting that it cannot be doped under normal conditions. Dopants exhibiting endoergic solution enthalpies can be doped experimentally to prepare [Na_3_(Fe_x_M_1−x_)_2_(PO_4_)_3_; M = Sc, La, Gd and Y; x = 0–1] composites. Such composites may have the different chemical, electronic, and mechanical properties required for different purposes. Gaining knowledge on the exact composition requires experiments.

### 3.5. Tetravalent Doping

Both the Fe and P sites were considered for tetravalent dopants (Si, Ge, Ti, Sn, Zr, and C). It is possible to form Na vacancies by doping with M^4+^ ions on the Fe site. Such vacancies facilitate Na ion migration in this material. The following reaction equation was constructed:

(10) $${\mathrm{RO}}_{2}\;+\;2{\mathrm{Fe}}_{\mathrm{Fe}}^{X}\;+\;2{\mathrm{Na}}_{\mathrm{Na}}^{X}\;\to \;2R_{\mathrm{Fe}}^{•}\;+\;2V_{\mathrm{Na}}^{\prime }\;+\;2{\mathrm{Fe}}_{2}O_{3}\;+{\;\mathrm{Na}}_{2}O$$

**Figure 6:**
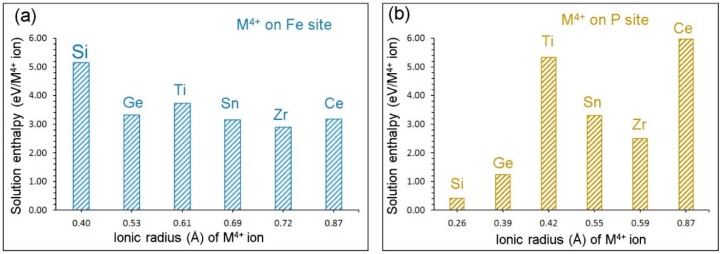
Solution enthalpies calculated for RO_2_ (R = Si, Ge, Ti, Sn, Zr and Ce) on (**a**) the Fe site and (**b**) the P site with respect to the M^4+^ radius in an octahedral and tetrahedral coordination, respectively.

Solution enthalpies are shown in Figure 6a. In all cases, high solution enthalpies (>3 eV) are observed, suggesting that the formation of Na vacancies are unlikely at normal temperatures. The lowest solution enthalpy is calculated for Zr. Solution enthalpies for Ge and Sn are very close to the value calculated for Zr. The Si exhibits the highest solution enthalpy.

Doping with M^4+^ ions on the P site can lead to the formation of Na interstitials, as shown in Equation (11). This engineering strategy in turn would enhance the capacity of Na_3_Fe_2_(PO_4_)_3_.

(11) $$2{\mathrm{RO}}_{2}\;+\;2P_{P}^{X}\;+{\;\mathrm{Na}}_{2}O\;\to \;2R_{P}^{\prime }\;+\;2{\mathrm{Na}}_{i}^{•}\;+{\;P}_{2}O_{5}$$

Solution enthalpies calculated for this process are shown in Figure 6b. The promising candidate is Si as its solution enthalpy is 0.41 eV. The Ge exhibits a slightly higher (1.23 eV) solution enthalpy. Other dopants have solution enthalpies greater than 2.50 eV, meaning that they should be doped at high temperatures. The highest solution enthalpy (5.98 eV) is calculated for Ce.

## 4. Conclusions

Atomistic simulation techniques were employed to examine the defects, Na ion migration pathways, and a variety of isovalent and isovalent dopants on the Fe site in Na_3_Fe_2_(PO_4_)_3_. The lowest energy defect process is the Na Frenkel, suggesting that the Na diffusion in this material would be assisted by Na vacancies. The Na-Fe anti-site defect is calculated to be the second lowest energy process, meaning that a small population of Na and Fe ions exchange their positions. The diffusion of Na is calculated to be low in this material and Na ion migrates via the *bc* plane with the activation energy of 0.45 eV. The favorable isovalent dopants on the Fe site are Sc, La, Gd, and Y, meaning that the synthesis of [Na_3_(Fe_x_M_1−x_)_2_(PO_4_)_3_ M = Sc, La, Gd and Y] is worth investigating experimentally. Doping with Zr on the Fe site can increase the concentration of Na vacancies needed for the Na ion diffusion, while doping with Si on the P site can facilitate the formation of Na interstitials required for the improvement in the capacity of Na_3_Fe_2_(PO_4_)_3_.

## Figures and Tables

**Table 1:** Comparison of experimental and calculated values of monoclinic (*C*2*/c*) Na_3_Fe_2_(PO_4_)_3_.

Parameter	Calc	Exp [48]	|∆| (%)
*a* (Å)	15.106	15.070	0.24
*b* (Å)	8.720	8.740	0.23
*c* (Å)	8.853	8.724	1.49
*α* (°)	90.0	90.0	0.00
*β* (°)	124.98	125.10	0.09
*γ* (°)	90.0	90.0	0.00

**Table 2:** Na-Na separations and their activation energies for the Na ion migration, as shown in Figure 3.

Migration Path	Na-Na Separation (Å)	Activation Energy (eV)
A	3.31	0.44
B	3.37	0.45
C	4.61	2.37

**Table 3:** Long-range Na ion diffusion paths and their overall activation energies.

Long-Range Path	Direction	Overall Activation Energy (eV)
A→A→B→B	*bc* plane	0.45
A→C→C→ A	*bc* plane	2.37
C→C→C→C	*bc* plane	2.37

## Supplementary Materials

The following are available online at https://www.mdpi.com/1996-1944/12/8/1348/s1: Table S1: Interatomic potential parameters used in the atomistic simulations of Na_3_Fe_2_(PO_4_)_3_.
