# Supplementary material for: Defect Chemistry and Na-Ion Diffusion in Na3Fe2(PO4)3 Cathode Material
Source: Materials (Basel). 2019 Apr 25;12(8):1348. doi: 10.3390/ma12081348 (PMC6515689; doi:10.3390/ma12081348)
Supplement: Supplementary file 1 [file materials-12-01348-s001.pdf]

materials

Supplementary Information

Defect Chemistry and Na-Ion Diffusion in $Na_3Fe_2(PO_4)_3$ Cathode Material

Navaratnarajah Kuganathan [1,2,*] and Alexander Chroneos [1,2]

[1] Department of Materials, Imperial College London, SW7 2AZ London, UK; alexander.chroneos@imperial.ac.uk
[2] Faculty of Engineering, Environment and Computing, Coventry University, Priory Street, CV1 5FB Coventry, UK
* Correspondence: n.kuganathan@imperial.ac.uk

Table S1. Interatomic potential parameters used in the atomistic simulations of $Na_3Fe_2(PO_4)_3$.

Two-Body [$\Phi_{ij}(r_{ij}) = A_{ij}\exp(-r_{ij}/\rho_{ij}) - C_{ij}/r_{ij}^6$]					
Interaction	A/eV	ρ/Å	C/eV·Å^6	Y/e	K/eV·Å^{-2}
Na^+–O^{2-} [1]	1497.830598	0.287483	0.00	1.000	99999
Fe^{3+}–O^{2-} [2]	1156.36	0.3299	0.00	4.970	304.7
O^{2-}–O^{2-} [3]	22764.30	0.149	27.89	–2.860	74.92
Al^{3+}–O^{2-} [4]	1114.9	0.2742	0.000	3.000	99999
Ga^{3+}–O^{2-} [5]	2901.12	0.2742	0.000	3.000	99999
Sc^{3+}–O^{2-} [4]	1299.4	0.3312	0.000	3.000	99999
Y^{3+}–O^{2-} [4]	1345.10	0.3491	0.000	3.000	99999
Gd^{3+}–O^{2-} [6]	1885.75	0.3399	20.34	3.000	99999
La^{3+}–O^{2-} [7]	1545.21	0.3590	0.000	-0.250	99999
Si^{4+}–O^{2-} [3]	1283.91	0.32052	10.66	4.000	99999
Ge^{4+}–O^{2-} [8]	1497.3996	0.325646	16.00	4.000	99999
Ti^{4+}–O^{2-} [9]	5111.7	0.2625	0.000	–0.100	314.0
Sn^{4+}–O^{2-} [10]	1414.32	0.3479	13.66	4.000	99999
Zr^{4+}–O2$^-$ [11]	985.869	0.3760	0.00	1.350	169.617
Ce^{4+}–O^{2-} [8]	1986.83	0.3511	20.40	7.700	291.75

References

1. Treacher, J.C.; Wood, S.M.; Islam, M.S.; Kendrick, E. Na_2CoSiO_4 as a cathode material for sodium-ion batteries: structure, electrochemistry and diffusion pathways. *Phys. Chem. Chem. Phys.* **2016**, *18*, 32744–32752.
2. Cherry, M.; Islam, M.S.; Catlow, C.R.A. Oxygen Ion Migration in Perovskite-Type Oxides. *J. Solid State Chem.* **1995**, *118*, 125–132.
3. Kuganathan, N.; Islam, M.S. Li_2MnSiO_4 Lithium Battery Material: Atomic-Scale Study of Defects, Lithium Mobility, and Trivalent Dopants. *Chem. Mater.* **2009**, *21*, 5196–5202.
4. Lewis, G.V.; Catlow, C.R.A. Potential models for ionic oxides. *J. Phys. C Solid State Phys.* **1985**, *18*, 1149.
5. Freeman, C.M.; Catlow, C.R.A. A computer modeling study of defect and dopant states in SnO_2. *J. Solid State Chem.* **1990**, *85*, 65–75.
6. Busker, G.; Chroneos, A.; Grimes, R.W.; Chen, I.-W. Solution Mechanisms for Dopant Oxides in Yttria. *J. Am. Ceram. Soc.* **1999**, *82*, 1553–1559.
7. Khan, M.S.; Islam, M.S.; Bates, D.R. Dopant Substitution and Ion Migration in the $LaGaO_3$-Based Oxygen Ion Conductor. *J. Phys. Chem. B* **1998**, *102*, 3099–3104.
8. Kuganathan, N.; Iyngaran, P.; Chroneos, A. Lithium diffusion in Li_5FeO_4. *Sci. Rep.* **2018**, *8*, 5832.
9. Olson, C.L.; Nelson, J.; Islam, M.S. Defect Chemistry, Surface Structures, and Lithium Insertion in Anatase TiO_2. *J. Phys. Chem. B* **2006**, *110*, 9995–10001.

10. Minervini, L.; Zacate, M.O.; Grimes, R.W. Defect cluster formation in M_2O_3-doped CeO_2. *Solid State Ion.* **1999**, *116*, 339–349.
11. Dwivedi, A.; Cormack, A.N. A computer simulation study of the defect structure of calcia-stabilized zirconia. *Philosophi. Maga. A* **1990**, *61*, 1–22.
